# Developing and refining the methods for a ‘one-stop shop’ for research evidence about health systems

**DOI:** 10.1186/1478-4505-13-10

**Published:** 2015-02-25

**Authors:** John N Lavis, Michael G Wilson, Kaelan A Moat, Amanda C Hammill, Jennifer A Boyko, Jeremy M Grimshaw, Signe Flottorp

**Affiliations:** McMaster Health Forum, 1280 Main St. West, MML-417, Hamilton, ON L8S 4L6 Canada; Centre for Health Economics and Policy Analysis, McMaster University, 1280 Main St. West, CRL-209, Hamilton, ON L8S 4K1 Canada; Department of Clinical Epidemiology and Biostatistics, McMaster University, 1280 Main St. West, CRL-209, Hamilton, ON L8S 4 K1 Canada; Department of Political Science, McMaster University, 1280 Main St. West, CRL-209, Hamilton, ON L8S 4 K1 Canada; Department of Global Health and Population, Harvard T.H. Chan School of Public Health, 677 Huntington Ave., Cambridge, MA 02115 USA; School of Health Studies, Western University, Arthur and Sonia Labatt Health Sciences Building, Room 403, London, ON N6A 5B9 Canada; Clinical Epidemiology Program, Ottawa Hospital Research Institute, The Ottawa Hospital – General Campus, 501 Smyth Rd., Box 711, Ottawa, ON K1H 8 L6 Canada; Department of Medicine, University of Ottawa, 451 Smyth Rd., Ottawa, ON K1H 8 M5 Canada; Norwegian Knowledge Centre for the Health Services, Boks 7004, St., Olavsplass, Oslo N-0130 Norway

## Abstract

**Background:**

Policymakers, stakeholders and researchers have not been able to find research evidence about health systems using an easily understood taxonomy of topics, know when they have conducted a comprehensive search of the many types of research evidence relevant to them, or rapidly identify decision-relevant information in their search results.

**Methods:**

To address these gaps, we developed an approach to building a ‘one-stop shop’ for research evidence about health systems. We developed a taxonomy of health system topics and iteratively refined it by drawing on existing categorization schemes and by using it to categorize progressively larger bundles of research evidence. We identified systematic reviews, systematic review protocols, and review-derived products through searches of Medline, hand searches of several databases indexing systematic reviews, hand searches of journals, and continuous scanning of listservs and websites. We developed an approach to providing ‘added value’ to existing content (e.g., coding systematic reviews according to the countries in which included studies were conducted) and to expanding the types of evidence eligible for inclusion (e.g., economic evaluations and health system descriptions). Lastly, we developed an approach to continuously updating the online one-stop shop in seven supported languages.

**Results:**

The taxonomy is organized by governance, financial, and delivery arrangements and by implementation strategies. The ‘one-stop shop’, called Health Systems Evidence, contains a comprehensive inventory of evidence briefs, overviews of systematic reviews, systematic reviews, systematic review protocols, registered systematic review titles, economic evaluations and costing studies, health reform descriptions and health system descriptions, and many types of added-value coding. It is continuously updated and new content is regularly translated into Arabic, Chinese, English, French, Portuguese, Russian, and Spanish.

**Conclusions:**

Policymakers and stakeholders can now easily access and use a wide variety of types of research evidence about health systems to inform decision-making and advocacy. Researchers and research funding agencies can use Health Systems Evidence to identify gaps in the current stock of research evidence and domains that could benefit from primary research, systematic reviews, and review overviews.

**Electronic supplementary material:**

The online version of this article (doi:10.1186/1478-4505-13-10) contains supplementary material, which is available to authorized users.

## Background

‘One-stop shops’ for research evidence can allow health system policymakers, stakeholders, and researchers to find and use the best available research evidence efficiently in the limited time they have available to make, inform, or advocate for a decision. Such resources have been highlighted as a central pillar of broader efforts to support evidence-informed decision-making about health systems [1]. One-stop shops are critical to ensuring that policymakers have timely access to research evidence when pressing issues emerge; this is one of the key factors found to increase the prospects for research use by policymakers [2]. However, while one-stop shops have been developed to address questions regarding clinical programs and services and prescription drugs [3–7], as well as to address questions regarding public health programs and services [8, 9], no similar resource existed for questions about health systems (i.e., how we strengthen healthcare and public health systems or how we get cost-effective healthcare and public health programs and services, as well as drugs and other technologies, to those who need them).

Developing a one-stop shop for health system policymakers, stakeholders, and researchers requires addressing three challenges. First, these groups need to be able to find research evidence about health systems using an easily understood taxonomy of topics. Such a taxonomy would ideally be organized in a way that reflects the ways in which these groups think about health systems (i.e., by focusing on ‘policy levers,’ which can include both health system arrangements and implementation strategies) and using terminology they are familiar with. While a number of taxonomies exist [10], they tend either to lack the specificity needed to capture the many different types of policy levers that exist (e.g., World Health Organization (WHO) ‘building blocks of health systems’ taxonomy [11]) or the breadth of levers available in health systems (e.g., taxonomies focusing on particular health system domains such as human resources or pharmaceutical policy [12, 13]).

Second, health system policymakers, stakeholders, and researchers need to know when they have conducted a comprehensive search of the many types of research evidence relevant to them. Providing such reassurance with a single one-stop shop means ensuring that it incorporates the many types of research evidence needed to answer their questions and contains comprehensive inventories of each of these types of research evidence. Many existing one-stop shops address questions about the effectiveness and cost-effectiveness of policy options (through systematic reviews, single studies such as impact evaluations and economic evaluations, or both), but not questions about the policy problem (e.g., stakeholders’ views and experiences with the problem) or about many features of policy options (e.g., how and why options work) and implementation considerations (e.g., barriers to implementing a particular options) [14, 15]. Moreover, many existing one-stop shops do not contain comprehensive inventories and, as a result, users are left uncertain about how many other databases they should search to address the same question.

Third, these groups need to be able to rapidly identify decision-relevant information in the results of their search of a given one-stop shop. For systematic reviews, for example, a busy policymaker would ideally like to be able to quickly scan the search results to know whether the retrieved reviews are of high quality, whether the searches for research evidence were conducted recently, and whether the studies included in the reviews were conducted in their own health system or in health systems that share characteristics that are likely to influence the applicability of the findings to their own system [2]. The same policymaker would ideally also like to read a structured decision-relevant summary of a particular review of interest; however, eight different groups in the world now prepare such summaries and they are not available on a single site [14].

Our objective was to develop and refine the methods for an easily searched, comprehensive, free, one-stop shop for research evidence that could provide decision-relevant information about the many types of questions asked by policymakers, stakeholders, and researchers regarding health systems. To address this objective, we proceeded in four stages: i) developing a taxonomy of governance, financial, and delivery arrangements within health systems and of implementation strategies within health systems; ii) building content by identifying, selecting, and categorizing content and by adding value to that content; iii) expanding the types of content; and iv) continuously updating the resulting one-stop shop (Table 1).

**Table 1:** **Stages in the creation of the one-stop shop**

Stage	Activities	Activities by source	Outcomes
1 – Developing a taxonomy	• Developed and iteratively refined a taxonomy of health system topics during stage 1 of content building	**Taxonomy development**	• Prototype for a taxonomy
		• Drew on existing categorization schemes	• Included systematic reviews (n = 616)
		o System-wide schemes such as WHO’s “building blocks” [11]	o Reviews of effects* (n = 513)
		o Domain-specific schemes such as those related to human resources policy [13], pharmaceutical policy [12], and implementation strategies [7]	■ Cochrane reviews of effects (n = 184)
	• Began building a repository of systematic reviews and systematic review protocols	• Used it to code progressively larger bundles of systematic reviews and systematic review protocols and made adjustments as needed	o Reviews addressing questions other than effects (cumulative n = 103)
		**Preliminary content building (phase 1)**	• Included Cochrane protocols of reviews of effects (n = 64)
		• Searched Medline (OVID) from 1966 to September 2006 (n = 848 reviews requiring eligibility assessment and, if eligible, coding)	
		• Hand searched the Cochrane Database of Systematic Reviews (CDSR) up to Issue 3, 2007 (n = 3,308 reviews and review protocols requiring eligibility assessment and, if eligible, coding)	
2 – Building content and adding value to that content	• Expanded breadth of searches	**Content building (phase 2)**	• Current taxonomy and cross-cutting taxonomy (Table 2)
	• Expanded scope of documents to include review-derived products	• Hand searched key databases and journals	• Comprehensive set of:*
		o CDSR for overviews of systematic reviews, systematic reviews and systematic reviews in progress (i.e., protocols) up to issue 7, 2012 and monitored each issue thereafter	o Systematic reviews (n = 4,240):
	• Iteratively refined the taxonomy during phase 2 of content building and complemented it with a cross-cutting taxonomy	o Database of Abstracts of Reviews of Effects (DARE) for systematic reviews up to April 2012 and monitored CRD News or a direct feed thereafter	■ Reviews of effects (n = 3,378 of which 585 are Cochrane reviews)
		o Rx for Change up to 2010 and monitored annual or semi-annual updates thereafter	■ Reviews addressing questions other than effects (n = 862)
		o Cochrane Qualitative and Implementation Methods Group’s reference database for qualitative reviews up to July 2012 and monitored it regularly thereafter	o Systematic reviews in progress (n = 423)
	• Began providing added value for existing content (e.g., for systematic reviews: assessing their methodological quality, coding them according to how recently searches were conducted and the countries in which included studies were conducted, and linking them to structured decision-relevant summaries)	o 15 journals for qualitative reviews from the first issue of 2004 to the last issue of 2008** (but hand searching not continued thereafter because of overlap with other sources)	o Review-derived products (n = 183)
		• Continuous scanning of listservs	■ Overviews of systematic reviews (n = 58)
		o Evidence Updates and KT+ for systematic reviews identified by McMaster PLUS	■ Evidence briefs (n = 125)
		o Listservs administered by PAHO EQUIDAD, PHCRIS, Sax Institute among	• ‘Added value’ coding available for all existing content
		• Review of websites for systematic reviews and reviewed-derived products (unless otherwise noted) up to June 2012 then continuous scanning	
		o 3Ie (International Initiative for Impact and Evaluation)	
		o Alliance for Health Policy and Systems Research	
		o Campbell Collaboration	
		o Canadian Institutes of Health Research ‘Evidence on Tap’ and ‘KT Synthesis’ programs	
		o Department for International Development (UK)	
		o Evidence for Policy and Practice Information and Coordinating Centre (EPPI-Centre)	
		o Evidence-Based Practice Centers (EPC), Agency for Healthcare Research and Quality (AHRQ)	
		o Evidence-Informed Policy Networks for evidence briefs [18]	
		o McMaster Health Forum for evidence briefs	
		o WHO Regional Office for Europe/Health Evidence Network/European Observatory on Health Systems and Policies for evidence briefs	
3 – Expanding the types of content	• Expanded scope of documents to include:	**Content building – phase 3**	• Comprehensive set of:*
		• Hand searched CDSR up to issue 10, 2012 of the CDSR for systematic reviews being planned (i.e., registered titles) and monitored each issue thereafter	o Systematic reviews being planned (n = 220)
	o Systematic reviews being planned		o Health reform descriptions (n = 1,107)
	o Economic evaluations and costing studies	• Hand searched two databases maintained by the Centre for Reviews and Dissemination	o Health system descriptions (n = 232)
	o Health reform descriptions	o PROSPERO up to October 2012 for systematic reviews being planned, and monitor it regularly thereafter	o Economic evaluations and costing studies published since 2003 (n = 2,236)
	o Health system descriptions	o Economic Evaluation Database up to October 2012 for economic evaluations and costing studies and monitored it regularly thereafter	• Partial (and soon to be comprehensive) set of links to studies included in systematic reviews, with the links assigned names based on the countries where the studies were conducted to facilitate immediate access to more locally applicable studies (91% of reviews now have links)
	• Providing more added value for existing content (e.g., links to studies included in systematic reviews)	• Hand searched Health Policy Monitor up to October 2012 for descriptions of health system reforms and monitored it regularly thereafter	
		• Hand searched the websites of the European Observatory on Health Systems and Policies, World Health Organization headquarters and all regional offices and World Bank up to October 2012 for health system descriptions and monitored them regularly thereafter	
4 – Continuously updating the one-stop shop	• Refined and executed procedures for continuously updating the one-stop shop in all seven supported languages	• Continue to monitor sources from the above stages, assess eligibility, categorize them, and add value to the content	• Procedures document for maintenance (with excerpts available upon request)
		• Continue to identify new potential sources and more efficient ways of accessing these sources, as well as new potential types of documents	• Agreements with partners to ensure the one-stop shop is functional in Arabic, Chinese, English, French, Portuguese, Russian and Spanish

*All numbers in this list are cumulative and include the documents identified in the previous stage.

**We hand searched three journals that had published two or more qualitative reviews of which we were already aware, namely the International Journal of Nursing Studies, Journal of Advanced Nursing, and Patient Education and Counseling. We also hand searched 12 journals that had published two or more policy-relevant reviews of any type, namely the American Journal of Managed Care, Evidence-based Healthcare & Public Health, Health Expectations, Health Policy, Health Policy & Planning, International Journal for Quality in Health Care, International Journal of Technology Assessment in Health Care, Journal of Health Services Research & Policy, Medical Care, Medical Care Research and Review, Psychiatric Services, and Social Science & Medicine.

## Methods

### Developing a taxonomy

We developed a taxonomy of health system topics and iteratively refined it by drawing on existing categorization schemes and by using it to categorize progressively larger bundles of systematic reviews and systematic review protocols. First, we drew on system-wide categorization schemes, such as the WHO’s ‘building blocks of health systems’ [11], and on domain-specific schemes such as those related to human resources policy, pharmaceutical policy, and implementation strategies [6, 7, 12, 13]. For example, we gained insights on governance arrangements from WHO’s ‘leadership and governance’ category; insights regarding financial arrangements from WHO’s ‘health financing’ category, and from the non-clinical (i.e., financial) aspects of WHO’s ‘medical products and technologies’ category (however, we also included four other sub-categories of financial arrangements that were not covered by the WHO framework). Further, we also obtained insights about delivery arrangements from WHO’s ‘service delivery’ category and WHO’s ‘health workforce’ category. We considered WHO’s ‘information and evidence’ category to relate to many sub-categories in our taxonomy.

Second, two reviewers independently used the taxonomy to categorize systematic reviews and systematic review protocols that were identified by searching Medline (OVID) and by hand searching the Cochrane Database of Systematic Reviews. The Medline search strategy was based on the one used by Cochrane’s Effective Practice and Organization of Care review group, which is the review group focused specifically on producing reviews about health system arrangements and implementation strategies [16]. We iteratively refined the taxonomy – adjusting its organization and terminology – as the reviewers coded progressively larger bundles of systematic reviews and systematic review protocols and identified issues warranting consideration. For example, we merged one set of second-level categories (‘with what information and communication technology is care provided’ and ‘with what level of quality and safety is care provided’) under the new heading ‘with what supports is care provided’. We also changed the second-level category ‘to whom care is provided and with what efforts to reach them’ to the more descriptive wording of ‘how care is designed to meet consumers’ needs’.

The taxonomy continues to be a ‘living document’ that we update periodically and then use to retrospectively re-categorize all records affected by a change in categorization (Table 2 and Additional file 1). The taxonomy is supported by a glossary containing definitions and synonyms for all categories and sub-categories and by a set of inclusion criteria for each document type. For example, our inclusion criteria for systematic reviews include a systematic search of multiple literature databases and explicit selection criteria.

**Table 2:** **Taxonomy of health-system topics and cross-cutting domains**

Category	Sub-categories
Health system topics (for third-level headings, see Additional file 1)	**Governance arrangements**
	Policy authority
	Organizational authority
	Commercial authority
	Professional authority
	Consumer & stakeholder involvement
	**Financial arrangements**
	Financing systems
	Funding organizations
	Remunerating providers
	Purchasing products and services
	Incentivizing consumers
	**Delivery arrangements**
	How care is designed to meet consumers’ needs
	By whom care is provided
	Where care is provided
	With what supports is care provided
	**Implementation strategies**
	Consumer-targeted strategy
	Provider-targeted strategy
	Organization-targeted strategy
Diseases	**Infectious diseases**
	HIV
	Tuberculosis
	Malaria
	Diarrheal disease
	Lower respiratory infections
	**Non-communicable diseases**
	Cancer
	Cardiovascular disease
	Diabetes
	Alzheimer and other dementias
	Chronic obstructive pulmonary
	**Other**
	Maternal and child health
	Accidents
	Mental health and addictions
Technologies	Drugs
	Devices
	Diagnostics
	Surgery
Sectors	Primary care
	Home care
	Hospital care
	Rehabilitation
	Long-term care
	Public health
Providers	Physician
	*Generalist*
	*Specialist*
	Nurse
	Pharmacist
	Allied health professional
	Lay/community health worker

### Building content and adding value to that content

We identified additional systematic reviews and systematic review protocols, as well as review-derived products (evidence briefs for policy and overviews of systematic reviews) [17, 18], through an additional hand search of the Cochrane Database of Systematic Reviews, hand searches of the Database of Abstracts of Reviews of Effects, Rx for Change, a database of qualitative reviews, and 15 journals likely to contain qualitative reviews, as well as continuous scanning of listservs and websites (Table 1). Two reviewers independently conducted all eligibility assessments and categorizations using the taxonomy and disagreements were resolved by consensus or, when that was not possible, by a third reviewer. While we found relatively high inter-rater reliability scores early in the process of building content [19], and we provide ongoing training to the reviewers, we have continued to use two reviewers in each step to reduce the chance of error given the large number of staff involved.

While building content for the one-stop shop and drawing on user testing and team member experiences in training and interacting with policymakers and stakeholders, we continued to iteratively refine the taxonomy (Table 2 and Additional file 1, with the categories that were added in the last round of revision marked in the supplemental file with an asterisk) and we complemented it with a taxonomy of cross-cutting domains that are organized by diseases, technologies, sectors, and providers (Table 2). We derived the list of diseases from WHO’s top 10 causes of death by broad income group [20], grouped them by communicable diseases, non-communicable diseases, and other conditions, and grouped others within these broad categories where appropriate (e.g., all cancers were grouped together and all causes of death related to maternal and child health were grouped together). We derived the groupings of technologies from previous work [21], and the groupings of sectors and providers based on our knowledge of health systems.

We also developed an approach to providing added value to existing content. For each systematic review, two reviewers independently assessed its methodological quality using AMSTAR [22], coded it according to how recently searches were conducted and to the countries in which included studies were conducted, and linked it to as many structured decision-relevant summaries as are available [14], scientific abstracts (e.g., in Medline), and freely available full text, if applicable. While AMSTAR was originally developed in a context where the focus was systematic reviews of effects, we are not aware of an alternative for systematic reviews addressing non-effectiveness questions and AMSTAR has been found to perform well for reviews of observational studies [23]. For each document of another type, two independent reviewers coded it according to the year it was published and the countries that are the focus of the document, and link it to scientific abstracts and freely available full text if applicable. Each document was also coded according to whether it has a low- and middle-income country (LMIC) focus, which includes LMIC(s) being the target of the document, including at least one author from an LMIC, and (for systematic reviews) including at least one study conducted in an LMIC.

### Expanding the types of content

In the third stage, we developed an approach to expanding the types of evidence eligible for inclusion (Table 1). The new types of eligible documents include systematic reviews being planned (i.e., registered titles for systematic reviews to assist with identifying what new syntheses are in an early stage of preparation), economic evaluations and costing studies (to assist with assessments of value for money), health reform descriptions (a description of what was done in a given reform process, how and why to give practical insight to those considering similar reforms), and health system descriptions (a description of a country’s health system, including key health system arrangements, to assist with local applicability assessments when research was conducted in another health system). We identified these new types of evidence from hand searches of: i) Cochrane Database of Systematic Reviews for systematic reviews being planned; ii) PROSPERO for systematic reviews being planned; iii) Economic Evaluation Database for economic evaluations and costing studies; iv) Health Policy Monitor for descriptions of health system reforms; and v) European Observatory on Health Systems and Policies, WHO headquarters and all regional offices, and World Bank for health system descriptions. We also added a new approach to adding value to existing content, namely inserting links to each of the studies in systematic reviews and naming each link according to the country where the study was conducted.

### Continuously updating the one-stop shop

**Figure 1:**
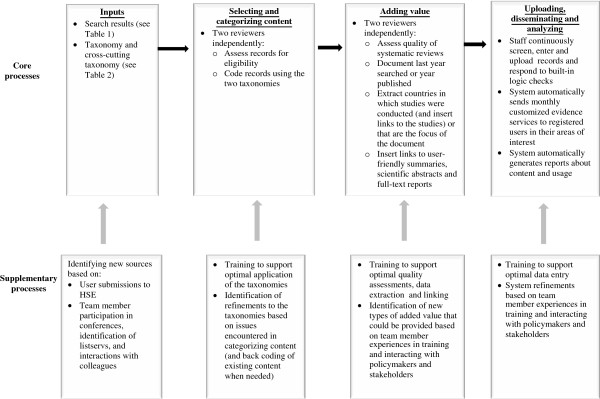
**Process for maintaining Health Systems Evidence.**

Finally, we developed an approach to continuously updating the online ‘one-stop shop’ in all six WHO official languages (Arabic, Chinese, English, French, Russian, and Spanish) as well as Portuguese (Table 1, Figure 1) and, through user testing, training workshops and other interactions with our target audiences, continuously adapting it based on their input. We continue to monitor all of the sources used in previous stages, assess eligibility, categorize them, and add value to the content. We also continue to identify new potential sources of documents and more efficient ways to access these sources, such as direct feeds. Finally, we remain open to suggestions for new types of documents that could address the questions being asked by health system policymakers, stakeholders, and researchers both within and across countries. For example, in Canada, these groups wanted to be able to access policy-relevant documents that provided key contextual information about the problems, policy options, and implementation issues being considered. We were able to rapidly develop an approach to identifying, categorizing, and coding these documents, integrating them into the one-stop shop, and making them visible to users registered as being based in Canada or who are accessing Health Systems Evidence from an IP address in Canada [24]. We also identified a similarly complementary set of policy-relevant documents about health system strengthening prepared by international agencies. The Intergovernmental Organizations’ Health Systems Documents Portal allows users to look for research evidence in the domains covered by 22 types of international agency documents, including World Health Assembly resolutions and WHO guidance.

## Results

The methodological developments described in this paper have led to the creation of Health Systems Evidence (http://www.healthsystemsevidence.org) – a one-stop shop for research evidence to support policymakers, stakeholders, and researchers interested in how to strengthen or reform health systems or in how to get cost-effective programs, services, and drugs to those who need them. The one-stop shop contains many functionalities designed to address the three challenges described earlier in this paper: health system policymakers, stakeholders, and researchers can now find research evidence about health systems using an easily understood taxonomy of topics, know when they have conducted a comprehensive search of the many types of research evidence relevant to them, and rapidly identify decision-relevant information in the results of their search (Table 3).

**Table 3:** **Current functionalities contained in Health Systems Evidence**

Domain	Functionality
Content	• Comprehensive inventory of the following types of research evidence about health system arrangements and implementation strategies
	o Evidence briefs for policy
	o Overviews of systematic reviews
	o Systematic reviews of effects
	o Systematic reviews addressing other questions
	o Systematic reviews in progress (i.e., protocols)
	o Systematic reviews being planned (i.e., registered titles)
	o Economic evaluations and costing studies
	o Health reform descriptions
	o Health system descriptions
Interface	• Available in all WHO official languages plus one other language (which collectively are spoken as the first or second language by a large proportion of the world’s population):
	o Arabic
	o Chinese
	o English
	o French
	o Portuguese
	o Russian
	o Spanish
Registration	• Free registration process enables users to sign up to receive a monthly customizable evidence service and to access complementary content (see below)
Open search	• Auto-complete feature operates with a synonyms dictionary
Advanced search	• Taxonomy can be expanded and sub-elements ticked for a highly specific search
	• Search terms can be entered and searched for in a variety of fields (e.g., title and abstract fields)
	• A number of limits can be applied to further refine the search, such as:
	o Taxonomy of cross-cutting domains
	Type of document (e.g., systematic review or economic evaluation and costing studies)
	o Publication date range
	o Country focus
	o Low- and middle-income country focus
Search results overview	• Overview of the types of research evidence available reminds users of the many types of evidence that they may want to consider (and highlights the type that they had selected in their search limits, if applicable)
	• Provides two types of additional search options if the search failed to retrieve relevant records:
	o Search for high-quality studies published since 2003 (and captured in Evidence Updates)
	o Search for studies published in Medline (and captured through a validated search strategy for types of research evidence particularly relevant to health system policymakers, stakeholders and researchers)
Search results by record	• Table allows for easy scanning by key features:
	o Title of record
	o Type of document
	o Last year literature searched (for reviews) or year published
	o Quality rating
	o Countries in which studies were conducted or that are the focus of the document
	• Table provides two ways to see more detail:
	o Links to structured decision-relevant summaries (with up to eight available depending on the record), abstracts, full-text reports, studies included in a systematic review (if applicable), and related documents (i.e., other types of research evidence on the same topic)
	o One-pager (see below)
One-pager	• One-pager provides a complete summary of all information available for the record, including the full citation and related documents
Additional resources	• Background information includes ‘why use it and who’s behind it,’ ‘what’s in it,’ and a four-page PDF about it
	• Search tips include ‘how to search it,’ ‘what a search will retrieve,’ and a description of any newly added content or functionality
	• Related tools include a one-page PDF on ‘finding and using research evidence’ and a two-page PDF containing links to the full suite of SUPPORT Tools for evidence-informed Policymaking (STP) [15]
	• Videos include a brief video about using Health Systems Evidence (currently available in select languages only)
Supplementary content	• For those registered as based in Canada or those who elect at registration to access additional content relevant to Canada, the Evidence-Informed Healthcare Renewal Portal is visible and can be searched for any policy-relevant documents related to health systems in Canada
	• The Intergovernmental Organizations’ Health Systems Documents Portal can be searched for policy-relevant documents about health system strengthening prepared by international agencies
	• Over time, additional supplementary content will be added

The taxonomy at the center of Health Systems Evidence is organized by governance, financial, and delivery arrangements and by implementation strategies (Table 2), and it is complemented by a taxonomy of the cross-cutting domains of diseases, technologies, sectors, and providers (Table 2). The one-stop shop now contains a comprehensive inventory of evidence briefs, overviews of systematic reviews, systematic reviews, systematic review protocols, registered systematic review titles, economic evaluations and costing studies, health reform descriptions, and health system descriptions, as well as many types of added-value coding (Table 1, last column). It is continuously updated using a variety of regularly scheduled hand searches, direct feeds, and other approaches and regular translation of new content into Arabic, Chinese, English, French, Portuguese, Russian, and Spanish (Figure 1). We provide a complete description of the contents of Health Systems Evidence in a separate paper [25].

## Conclusions

Policymakers and stakeholders can now easily access and use a wide variety of types of research evidence about health systems to inform decision-making and advocacy. Moreover, they can also access and use complementary content, such as WHO policy-relevant documents. Researchers and research funding agencies can use Health Systems Evidence to identify gaps in the current stock of research evidence and domains that could benefit from primary research, systematic reviews and overviews of reviews.

The main strengths of our approach include: i) developing and iteratively refining a taxonomy based on existing categorization schemes and the practical challenges that arise when using it to code a heterogeneous body of research evidence; ii) building content through systematic searches of a broad array of sources, adding value to that content in ways that have been identified as important to policymakers and stakeholders, and having two reviewers independently participate in each step of the process; iii) expanding the types of content included in the one-stop shop as we achieve comprehensiveness for existing content and recognize the next most pressing gap faced in the type of research evidence needed; iv) designing the one-stop shop in a way that uses links to drive traffic to the websites of those groups involved in producing and/or disseminating these documents (e.g., Cochrane Library, Centre for Reviews and Dissemination, Rx for Change) or in preparing decision-relevant summaries (e.g., Australasian Cochrane Centre and SUPPORT collaboration) so that these groups get credit for their work in the form of website hits; and v) continuously updating the one-stop shop in all seven supported languages.

The limitations of our study are: i) having some sub-categories in the taxonomy containing very large numbers of documents, which could frustrate busy policymakers, stakeholders, and researchers; ii) potentially missing review-derived products, particularly evidence briefs [17], which are a new type of research product and not easily found through traditional search mechanisms; and iii) having a user interface that can appear overly complicated to new users. We are now working to address these limitations, particularly the user interface by engaging in ongoing user testing with health system policymakers and stakeholders.

## Electronic supplementary material

Additional file 1: **Taxonomy of governance, financial and delivery arrangements within health systems and of implementation strategies within health systems.** (DOCX 18 KB)

## Abbreviations

LMIC: Low- and middle-income country
WHO: World Health Organization.

## References

[1] Lavis JN, Lomas J, Hamid M, Sewankambo NK (2006). Assessing country-level efforts to link research to action. Bull World Health Org.

[2] Lavis JN, Davies HTO, Oxman AD, Denis J-L, Golden-Biddle K, Ferlie E (2005). Towards systematic reviews that inform health care management and policy-making. J Health Serv Res Policy.

[3] BMJ Evidence Centre (2013). Evidence updates.

[4] Haynes RB (2005). bmjupdates+, a new FREE service for evidence-based clinical practice. Evid Based Nurs.

[5] Cochrane Collaboration (2013). The Cochrane Library.

[6] Canadian Agency for Drugs and Technologies in Health (2013). CADTH: Rx for Change.

[7] Weir M, Ryan R, Mayhew A, Worswick J, Santesso N, Lowe D (2010). The Rx for Change database: a first-in-class tool for optimal prescribing and medicines use. Implement Sci.

[8] Dobbins M, DeCorby K, Robeson P, Husson H, Tirilis D, Greco L (2010). A knowledge management tool for public health: health-evidence.ca. BMC Public Health.

[9] Health-Evidence.ca (2013). Health evidence.

[10] Hoffman SJ, Røttingen JA, Bennett S, Lavis JN, Edge J, Frenk J (2012). Background paper on conceptual issues related to health systems research to inform a WHO global strategy on health systems research.

[11] Alliance for Health Policy and Systems Research (2009). What is health policy and systems research and why does it matter?.

[12] Aaserud M, Dahlgren AT, Sturm H, Kösters JP, Hill S, Furberg CD (2006). Pharmaceutical policies: effects on rational drug use, an overview of 13 reviews. Cochrane Database Syst Rev.

[13] Chopra M, Munro S, Lavis JN, Vist G, Bennett S (2008). Effects of policy options for human resources for health: an analysis of systematic reviews. Lancet.

[14] Lavis JN (2009). How can we support the use of systematic reviews in policymaking?. PLoS Med.

[15] Lavis JN, Oxman AD, Lewin SA, Fretheim A (2009). Introduction: SUPPORT Tools for evidence-informed health Policymaking (STP). Health Res Policy Syst.

[16] McAuley LM, Grimshaw J, Zwarenstein M (2003). Scope of EPOC is clarified. BMJ.

[17] Lavis JN, Permanand G, Oxman AD, Lewin SA, Fretheim A (2009). SUPPORT Tools for evidence-informed health Policymaking (STP) 13: preparing and using policy briefs to support evidence-informed policymaking. Health Res Policy Syst.

[18] Lavis JN, Panisset U (2010). EVIPNet Africa’s first series of policy briefs to support evidence-informed policymaking. Int J Technol Assess Health Care.

[19] Lavis JN, Wilson MG, Burchett H, Grimshaw JM, Haynes RB, Oxman AD (2008). Making Cochrane reviews more accessible to policymakers.

[20] World Health Organization (2008). The 10 leading causes of death by broad income group.

[21] Lavis JN, Wilson MG, Grimshaw JM, Haynes RB, Ouimet M, Raina P (2010). Supporting the use of health technology assessments in policy making about health systems. Int J Technol Assess Health Care.

[22] Shea B, Grimshaw J, Wells G, Boers M, Andersson N, Hamel C (2007). Development of AMSTAR: a measurement tool to assess the methodological quality of systematic reviews. BMC Med Res Methodol.

[23] Pieper D, Mathes T, Eikermann M (2014). Can AMSTAR also be applied to systematic reviews of non-randomized studies?. BMC Res Notes.

[24] Kowalewski K (2012). Mobilizing the use of policy-relevant documents in evidence-informed health policymaking: the development and contents of an online repository of policy-relevant documents addressing healthcare renewal in Canada.

[25] Wilson MG, Moat KA, Lavis JN (2013). The global stock of research evidence relevant to health systems policymaking. Health Res Policy Syst.

