# Supplementary material for: Developing and refining the methods for a ‘one-stop shop’ for research evidence about health systems
Source: Health Res Policy Syst. 2015 Feb 25;13:10. doi: 10.1186/1478-4505-13-10 (PMC4429608; doi:10.1186/1478-4505-13-10)
Supplement: Supplementary file 1 — Additional file 1: Taxonomy of governance, financial and delivery arrangements within health systems and of implementation strategies within health systems. (DOCX 18 KB) [file 12961_2014_379_MOESM1_ESM.docx]

**Supplemental file:**  **Taxonomy of governance, financial and delivery arrangements within health systems and of implementation strategies within health systems**

| **Governance arrangements** | **Financial arrangements** | **Delivery arrangements** | **Implementation strategies** |
| --- | --- | --- | --- |
| *Policy authority*  - Centralization/decentralization of policy authority - Accountability of the state sector’s role in financing & delivery* - Stewardship of the non-state sector’s role in financing & delivery - Decision-making authority about who is covered and what can or must be provided to them - Corruption protections  *Organizational authority*  - Ownership - Management approaches* - Accreditation - Networks/multi-institutional arrangements  *Commercial authority*  - Licensure & registration requirements - Patents & profits - Pricing & purchasing - Marketing - Sales & dispensing - Commercial liability  *Professional authority*  - Training & licensure requirements - Scope of practice - Setting of practice - Continuing competence - Quality & safety - Professional liability  *Consumer & stakeholder involvement*  - Consumer participation in policy & organizational decisions - Consumer participation in system monitoring* - Consumer participation in service delivery - Consumer complaints management - Stakeholder participation in policy & organizational decisions (or monitoring) | *Financing Systems*  - Taxation - Social health insurance - Community-based health insurance - Community loan funds* - Private insurance - Health savings accounts (Individually financed)* - User fees - Donor contributions*  *Funding organizations*  - Fee-for-service - Capitation - Global budget - Prospective payment - Indicative budgets - Targeted payments/penalties  *Remunerating providers*  - Fee-for-service - Capitation - Salary - Prospective payment - Fundholding* - Indicative budgets - Targeted payments/penalties - *Purchasing products & services* - Scope & nature of insurance plans* - Lists of covered/reimbursed organizations, providers, services & products - Restrictions in coverage/reimbursement rates for organizations, providers, services & products - Caps on coverage/reimbursement for organizations, providers, services & products - Prior approval requirements for organizations, providers, services & products - Lists of substitutable services & products - *Incentivizing consumers* - Premium (level & features) - Cost sharing - Health savings accounts (Third party contributions)* - Targeted payments/penalties | *How care is designed to meet consumers’ needs*  - Availability of care* - Timely access to care - Culturally appropriate care - Case management - Package of care/care pathways/disease management - Group care*  *By whom care is provided*  - System - Need, demand & supply - System - Recruitment, retention & transitions - System - Performance management - Workplace conditions – Provider satisfaction - Workplace conditions – Health & safety - Skill mix – Role performance - Skill mix – Role expansion or extension - Skill mix – Task shifting/substitution - Skill mix - Multidisciplinary teams - Skill mix – Volunteers* - Skill mix – Communication & case discussion between distant health professionals - Staff - Training - Staff - Support - Staff - Workload/workflow/intensity - Staff - Continuity of care - Staff/self – Shared decision-making - Self-management  *Where care is provided*  - Site of service delivery - Physical structure, facilities & equipment - Organizational scale - Integration of services - Continuity of care - Outreach*  *With what supports is care provided*  - Health record systems - Electronic health record - Other ICT that support individuals who provide care - ICT that support individuals who receive care - Quality monitoring and improvement systems - Safety monitoring and improvement systems | - *Consumer-targeted strategy* - Information or education provision - Behaviour change support - Skills and competencies development - (Personal) Support - Communication and decision-making facilitation - System participation - *Provider-targeted strategy* - Educational material - Educational meeting - Educational outreach visit - Local opinion leader - Local consensus process - Peer review - Audit and feedback - Reminders and prompts - Tailored intervention - Patient-mediated intervention - Multi-faceted intervention - *Organization-targeted strategy* |

*Categories that were added in the final phase of taxonomy development
